# Insights from serial cardiovascular magnetic resonance imaging show early progress in diastolic dysfunction relates to impaired right ventricular deformation

**DOI:** 10.1038/s41598-025-87032-5

**Published:** 2025-02-03

**Authors:** Sören J. Backhaus, Alexander Schulz, Torben Lange, Simon F. Rösel, Lennart S. Schmidt-Schweda, Shelby Kutty, Johannes T. Kowallick, Julia Treiber, Andreas Rolf, Samuel Sossalla, Gerd Hasenfuß, Andreas Schuster

**Affiliations:** 1 Department of Cardiology, Campus Kerckhoff of the Justus-Liebig-University Giessen, Kerckhoff-Clinic, Bad Nauheim, Germany; 2 German Center for Cardiovascular Research (DZHK), Partner Site Rhine-Main, Bad Nauheim, Germany; 3 Department of Cardiology and Angiology, Medical Clinic I, University Hospital Giessen, Justus-Liebig-University Giessen, Giessen, Germany; 4 Department of Cardiology and Pneumology, University Medical Center Göttingen, Georg-August University, Göttingen, Germany; 5 German Center for Cardiovascular Research (DZHK), Partner Site Lower Saxony, Göttingen, Germany; 6 Department of Medicine, Cardiovascular Division, Beth Israel Deaconess Medical Center, Harvard Medical School, Boston, USA; 7 Taussig Heart Center, Johns Hopkins Hospital, Baltimore, MD USA; 8 FORUM Radiology, Rosdorf, Germany; 9 FORUM Cardiology, An der Ziegelei 1, 37124 Rosdorf, Germany

**Keywords:** Heart failure, Risk factors

## Abstract

Latent pulmonary vascular disease is a distinct feature already in the early pathophysiology of masked heart failure with preserved ejection fraction (HFpEF) and associated with reduced right ventricular (RV) functional reserve. We hypothesized that serial real-time cardiovascular magnetic resonance (CMR) imaging at rest and during exercise-stress may detect early progress in pathophysiological alterations in HFpEF. Patients presenting with exertional dyspnoea and signs of diastolic dysfunction (E/e’>8, left ventricular (LV) ejection fraction > 50%) were prospectively enrolled in the HFpEF Stress Trial (NCT03260621). Rest and exercise-stress echocardiography, CMR and right heart catheterisation were performed at baseline. Pulmonary capillary wedge pressure (PCWP) was used for classification of HFpEF (≥ 15/25mmHg at rest/during exercise-stress) and non-cardiac dyspnoea (NCD). Repeat rest and exercise-stress CMR was performed in median 2.94 years after recruitment during which timeframe some HFpEF patients had undergone interatrial shunt device (IASD) implantation. Cardiovascular events were assessed after 4 years.Serial CMR scans were available for NCD *n* = 10, HFpEF *n* = 10 and HFpEF with IASD implantation following baseline diagnosis *n* = 6. RV long axis strain at rest and during exercise-stress decreased in HFpEF (*p* = 0.007 for both) but neither in NCD nor HFpEF with IASD. In contrast, in NCD, an improvement in LA LAS during exercise-stress (*p* = 0.028) was noted. There were no functional alterations in HFpEF patients who had undergone IASD implantation. RV functional deterioration may be a pathophysiological feature during early-stage disease progress in HFpEF. In this observational study RV functional deterioration was detected in HFpEF patients only but not patients with NCD and patients with HFpEF that were treated with IASD placement. These findings should next be explored in adequately powered future research trials. *Clinicaltrials.gov*: NCT03260621 (First posted date 24/08/2017).

## Introduction

The long-term registry of the European Association of Cardiology reports 40% of the heart failure (HF) population to be considered either amongst mildly reduced or preserved ejection fraction (HFmrEF/HFpEF) patients^1^. Notwithstanding the revolutionary introduction of SGLT-2 Inhibitors in HFpEF^2^ or an interatrial shunt device (IASD) for congestion relief associated with symptom severity^3,4^, all available strategies are united by their lack of mortality reduction. An underlying reason may be late therapeutic intervention in cardiac remodelling^5–7^ with potentially limited efficacy at later disease stages. Despite HFpEF being generally considered to be associated with slower disease progression and better survival^1^ available evidence indicates the presence of early remodelling processes and development of multiorgan disease including pulmonary vascular disease (PVD)^8^. Indeed, only patients without latent PVD as defined by a pulmonary vascular resistance (PVR) of 1.74 Wood units during exercise-stress right heart catheterisation (RHC) showed beneficial effects linked to IASD implantation^9^. This may potentially arise from preserved right ventricular (RV) function during early stages of disease only^10^.

We hypothesised that state-of-the-art cardiovascular magnetic resonance (CMR)^11,12^ imaging would identify signs of adverse remodelling in the early stage of HFpEF and discriminate pathophysiological differences between HFpEF, HFpEF treated with IASD and non-cardiac dyspnoea (NCD). Consequently, we initiated a follow-up CMR study of patients included in the HFpEF stress trial, who initially presented with exertional dyspnoea and had undergone RHC and CMR for detection and classification of HF to detect subtle changes in cardiac physiology.

## Methods

**Fig. 1:**
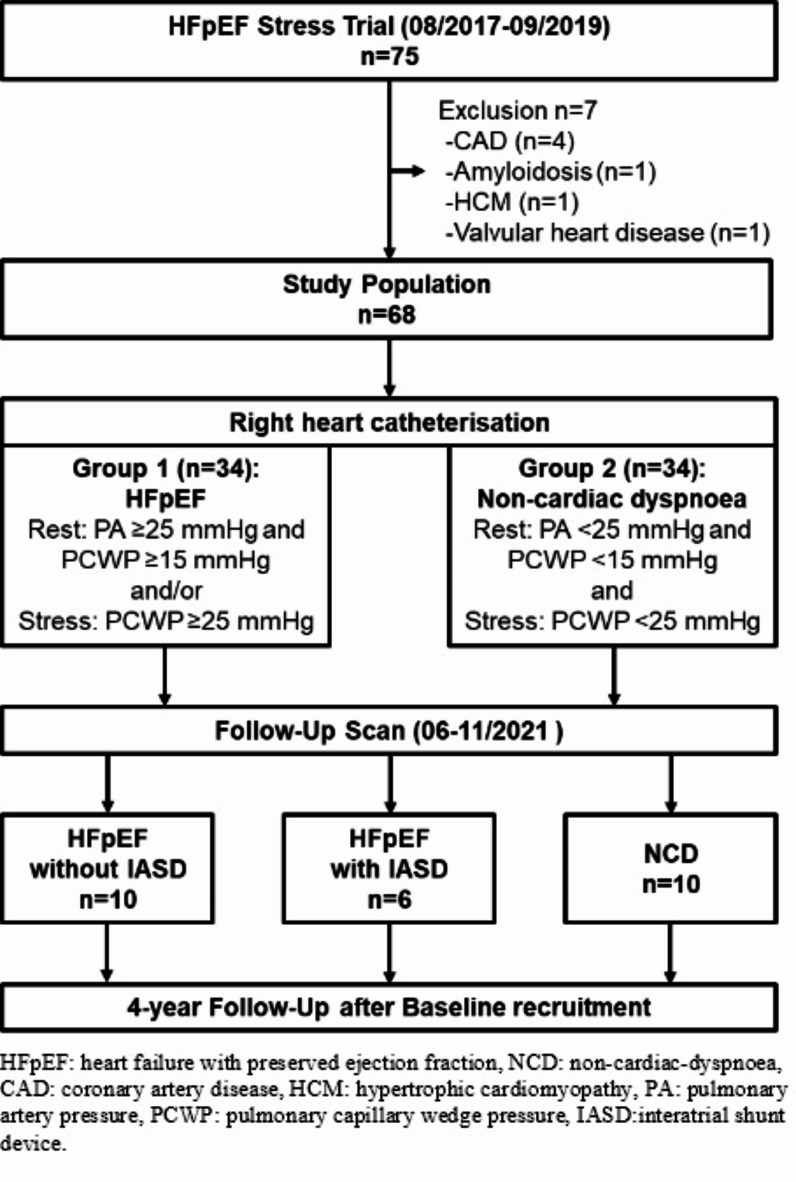
Study flow-chart.

The present study represent the clinical follow-up of the HFpEF Stress Trial (NCT03260621, first posted date 24/08/2017)^13^, Fig. 1. Briefly, the HFpEF Stress Trial prospectively recruited 75 patients with exertional dyspnoea (NYHA class ≥ II) and echocardiographic signs of diastolic dysfunction (E/e’ >8, EF > 50%) between 08/2017 and 09/2019. Exclusion criteria for study participation have been reported previously ^13^ and comprised known cardio-pulmonary disease associated with dyspnoea as well as common contraindications for CMR imaging^14^. At baseline, all patients underwent RHC as well as echocardiographic and CMR imaging at rest and during exercise-stress at an average heart rate of 100–110 beats/minute at a revolution of 50–60 rounds/minute on bicycle ergometry. NCD and HFpEF (PCWP at rest ≥ 15 mmHg and/or during exercise-stress ≥ 25mmHg on RHC) patients were approached for a follow-up survey between 06 and 11/2021. Follow-up examinations included laboratory testing, echocardiography at rest as well as CMR imaging at rest and during exercise-stress. Additionally, telephone interviews including review of medical records were conducted 4 years after baseline recruitment for the assessment of cardiovascular events (CVE)^15^. After initial study participation, some HFpEF patients were recruited to the Reduce LAP-HF II trial^9^ to receive an IASD. For overall clarity, these HFpEF patients are referred to as IASD patients although at baseline (initial study participation) the IASD had not yet been implanted.

### CMR imaging

The follow-up scan was conducted on the identical clinical 3.0 Tesla Magnetom Skyra MRI scanner (Siemens Healthcare, Erlangen, Germany).

**Fig. 2:**
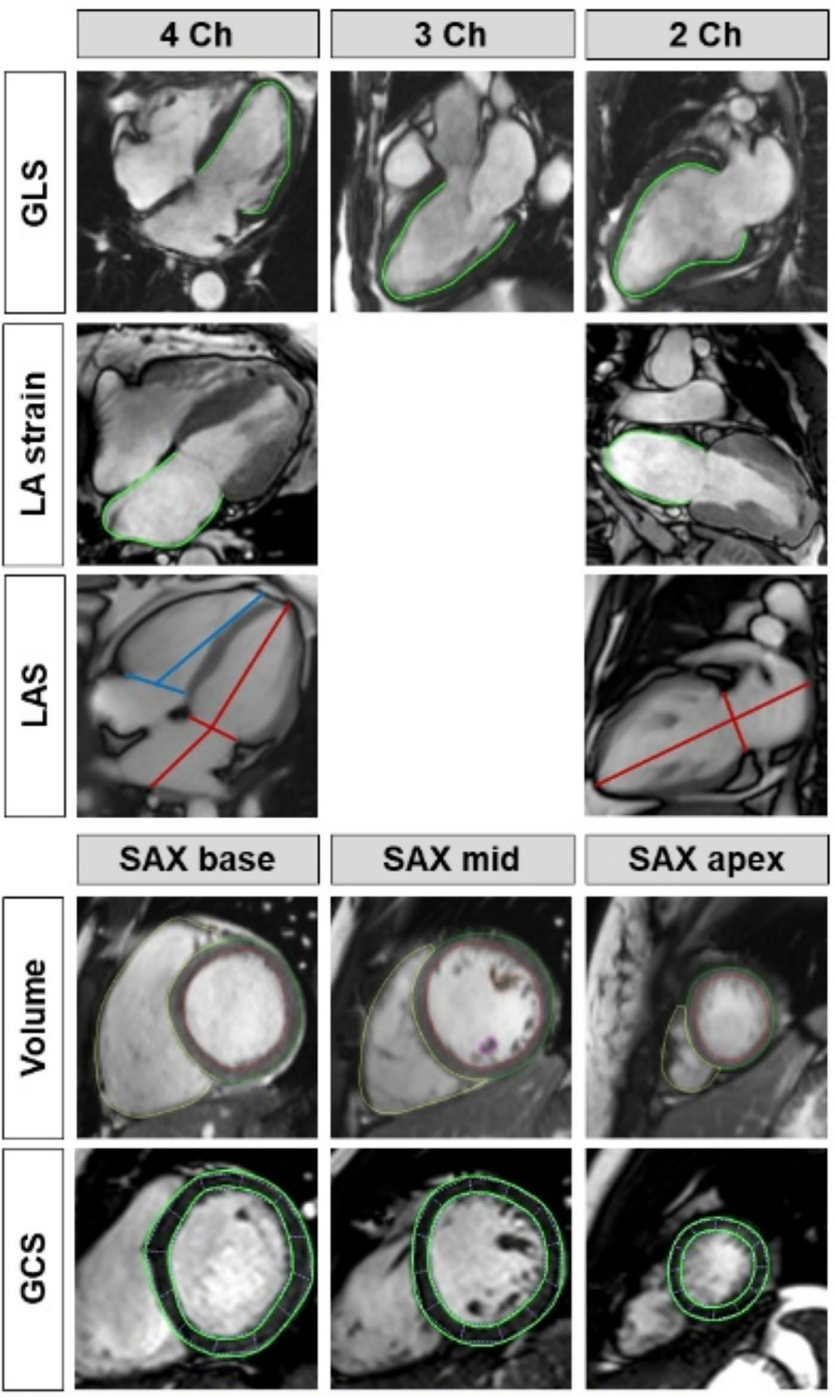
Cardiac functional quantification. Assessment of left ventricular global longitudinal strain (GLS), biventricular long axis strains (LAS) as well as left atrial (LA) strain on long axis chamber view (Ch) orientations. Ventricular volumes were acquired from a short axis (SAX) stack covering the entire ventricle (exemplary shown for 3 slices) which were used for global circumferential stain (GCS) evaluation.

Conventional imaging at rest was performed using steady state free precession (bSSFP) cine sequences for the acquisition of long axis (LAX) 2-, 3- and 4 chamber views (Ch) as well as a short axis (SAX) stack. Dedicated commercially available software Qmass/QStrain module provided by Medis, Medical Imaging Systems, Leiden, Netherlands was used for post-processing and comprised the following analyses, Fig. 2: Volumetric-based analyses consisted of left ventricular (LV) mass, LV/RV end-diastolic/systolic and stroke (EDV/ESV/SV) volumes as well as associated EF. Feature-tracking (FT) deformation was performed on all 4 cardiac chambers for the assessment of LV global longitudinal (GLS) and circumferential (GCS) strain. Left atrial (LA) function was classified according to reservoir function Es (collection of venous return), passive conduit function Ee (early ventricular filling) and active booster pump function Ea (late active augmentation of ventricular filling)^16–19^.

Real-Time free-breathing imaging was conducted at rest and during exercise-stress employing a strongly undersampled radial encoding scheme on a bSSFP sequence as described previously^20^. Cine sequences were acquired over several heart beats for LAX 2/4 Ch and a SAX stack. Post-processing was performed using OsiriX MD (Pixmeo SARL, CH-1233 Bernex, Switzerland), Fig. 2: Long axis strains (LAS) were assessed on LV/RV/LA cardiac chambers measuring the distance between the middle of a line connecting the origins of the mitral or tricuspid leaflets and epicardial apical LV/RV border or most distal wall of the LA respectively. LAS was calculated as follows^21,22^:

$$LV/RV LAS=\frac{Length_\text{enddiastole}-Length_\text{endsystole}}{Length_\text{enddiastole}}*100$$

$$LA \;LAS=\frac{Length_\text{endsystole}-Length_\text{enddiastole}}{Length_\text{endsystole}}*100$$

### Statistical analyses

Continuous variables are reported as median with associated interquartile ranges (IQR) and were compared using the Mann-Whitney U test if independent or the Wilcoxon signed-rank test if dependent. Overall differences in cardiovascular risk factors between groups were tested using the Kruskal–Wallis test. Predictors for cardiovascular events were evaluated using Cox regression models. A 2-tailed p-value < 0.05 was considered statistically significant. Calculations were performed using SPSS version 27.0 (IBM, Armonk, New York, USA) and MedCalc version 20.027 (MedCalc Software bvba, Ostend, Belgium).

## Results

### Study population

The follow-up population consisted of 10 NCD and 16 HFpEF patients, 6 of which had received an IASD following initial HFpEF diagnosis, Fig. 1. At baseline, there were no differences in cardiovascular risk factors including body mass index (p = 0.347), diabetes (p = 0.354), hypertension (p = 0.530), hyperlipidemia (p = 0.897), nicotine (p = 0.138) and sleep apnoea (p = 0.732). The follow-up scan was conducted in median 2.94 years (IQR 2.37, 3,27) after the initial baseline scan. At baseline laboratory testing revealed significantly increased NTproBNP in HFpEF who were (p = 0.002) or were not (p = 0.004) going to receive an IASD. A significant increase in NTproBNP from baseline to follow-up in NCD patients (p = 0.013) paralleled by a numerical increase in IASD patients (p = 0.917) resulted in maintained statistical difference at follow-up comparing NCD to IASD (p = 0.022) but not NCD to HFpEF (p = 0.105). There were no differences in echocardiographic findings for E/e’ and TAPSE at baseline or follow-up, Tables 1, 2, 3, 4 and 5.

**Table 1:** Cardiovascular magnetic resonance imaging baseline.

Variable	NCD baseline	HFpEF baseline	HFpEF IASD	NCD vs. HFpEF	NCD vs. IASD	HFpEF vs. IASD
**Laboratory testing**						
NTproBNP	55 (37, 111)	209 (80, 320)	230 (141, 998)	**0.004**	**0.002**	0.368
**Echocardiography**						
E/e’	9.6 (8.6, 12.0)	11.5 (10.3, 13.5)	12.8 (9.1, 12.9)	0.063	0.263	0.958
TAPSE	21.8 (19.1, 26.4)	23.6 (21.0, 28.8)	25.6 (23.1, 28.5)	0.447	0.145	0.562
**Right heart catheterisation**						
Pulmonary vascular resistance	1.48	1.71	1.05	0.218	0.263	**0.007**
**Left ventricle**						
LV Mass	55.3 (48.1, 72.2)	52.2 (47.9, 62.1)	60.0 (47.0, 67.5)	0.631	0.958	0.562
LV EDV	65.9 (54.9, 80.3)	65.3 (60.7, 75.7)	59.0 (51.6, 74.4)	0.853	0.562	0.263
LV ESV	19.6 (13.0, 23.3)	16.7 (14.3, 24.5)	17.1 (14.7, 26.4)	0.971	0.875	0.875
LV SV	45.1 (40.0, 59.2)	49.3 (44.3, 55.1)	44.6 (33.0, 51.0)	0.579	0.492	0.220
LV EF	73.4 (63.9, 77.2)	75.8 (66.5, 77.2)	71.6 (58.4, 75.8)	0.579	0.562	0.220
FT LV GLS	−26.6 (−23.4, −29.2)	−27.2 (−21.2, −30.4)	−23.4 (−20.8, −26.8)	0.912	0.220	0.313
FT LV GCS	−37.2 (−40.0, −34.1)	−37.4 (−32.2, −41.2)	−40.8 (−28.4, −43.5)	0.796	0.635	0.713
LV LAS rest	13.7 (13.1, 15.4)	13.8 (11.5, 15.5)	12.3 (7.5, 13.9)	1.000	0.093	0.181
LV LAS stress	17.8 (13.9, 20.7)	15.6 (12.9, 17.5)	13.6 (11.6, 17.0)	0.353	0.147	0.368
Septal Native T1	1314 (1288, 1335)	1302 (1264, 1334)	1344 (1289, 1395)	0.696	0.414	0.181
Septal ECV	24.9 (21.8, 26.9)	25.6 (24.4, 29.2)	25.0 (24.9, 27.0)	0.156	0.456	0.492
**Left atrium**						
FT LA Es	27.1 (20.7, 29.6)	22.8 (13.9, 24.6)	20.7 (14.4, 24.5)	0.075	0.073	0.875
FT LA Ee	12.2 (6.5, 17.2)	11.0 (6.5, 13.5)	8.5 (7.9, 12.7)	0.353	0.713	0.792
FT LA Ea	12.7 (11.2, 16.8)	10.5 (6.2, 13.5)	10.0 (6.2, 13.7)	0.105	0.118	1.000
LA LAS rest	21.0 (19.4, 23.3)	12.8 (8.5, 20.9)	12.1 (8.2, 16.4)	0.052	**< 0.001**	0.713
LA LAS stress	20.5 (15.7, 28.9)	18.3 (13.2, 20.5)	13.0 (8.9, 18.5)	0.247	0.073	0.263
**Right ventricle**						
RV EDV	64.6 (48.4, 76.6)	65.0 (58.1, 71.7)	65.1 (51.3, 81.4)	0.739	0.875	1.000
RV ESV	22.4 (16.1, 28.7)	20.0 (16.4, 23.0)	23.2 (16.9, 29.2)	0.529	0.792	0.562
RV SV	42.5 (34.8, 48.8)	45.6 (38.7, 51.3)	44.1 (28.9, 55.8)	0.393	1.000	0.792
RV EF	65.3 (60.7, 70.9)	69.5 (63.7, 74.9)	64.8 (60.2, 68.9)	0.218	0.875	0.263
RV LAS rest	18.3 (16.8, 22.2)	26.3 (20.9, 30.3)	18.0 (16.8, 20.9)	**0.015**	0.713	**0.011**
RV LAS stress	18.9 (15.1, 23.3)	24.2 (21.2, 27.3)	21.6 (19.2, 27.0)	0.052	0.263	0.428

TAPSE: tricuspid annular plane systolic excursion, LV: left ventricular, EDV/ESV: end-diastolic/-systolic volume, SV: stroke volume, EF: ejection fraction, FT: Feature-Tracking, GLS/GCS: Global longitudinal/circumferential strain, LAS: long axis strain, ECV: extracellular volume, LA: left atrium, Es/e/a: reservoir/conduit/booster pump function, RV: right ventricle. Bold p-values indicate statistical significance below 0.05. Volumes are given in ml/m² BSA, mass in g/m² BSA, strain/EF/ECV in %, T1 in ms, NTproBNP in pg/ml, TAPSE in mm and pulmonary vascular resistance in Wood units.

### Changes from baseline to follow-up in cardiac function

#### Baseline

At baseline, there were no differences in LV cardiac function comparing NCD, HFpEF and IASD patients. Compared to NCD, IASD patients showed significantly decreased LA LAS (*p* < 0.001) whilst there was a strong statistical trend in HFpEF (*p* = 0.052). This was paralleled by statistical trends for decreased LA Es in HFpEF (*p* = 0.075) and IASD (*p* = 0.073). HFpEF patients showed increased resting RV LAS compared to NCD (*p* = 0.015) and IASD (*p* = 0.011), Table 1.

### Baseline vs. follow-up

**Fig. 3:**
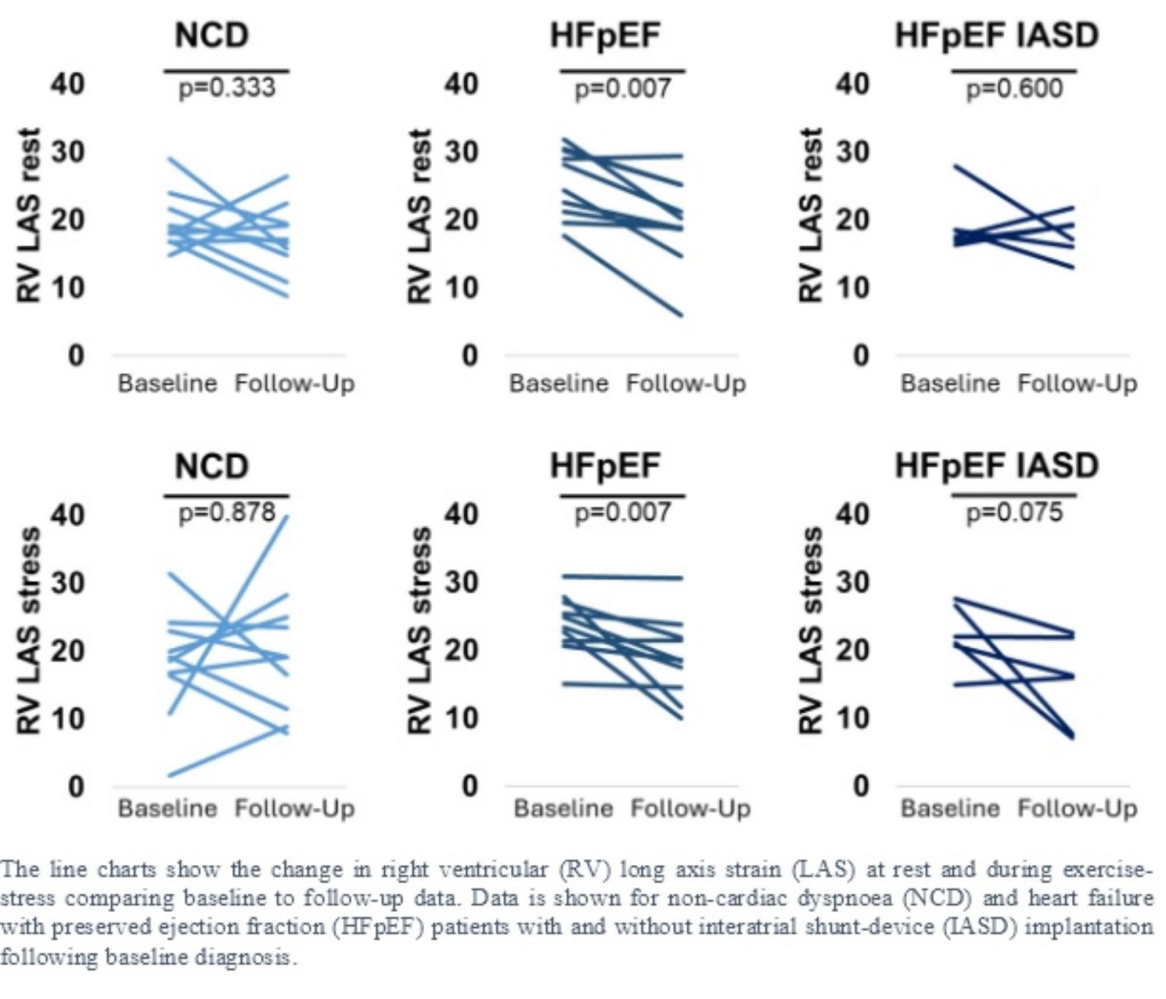
Change in right ventricular long axis strain.

Changes in cardiac function from baseline to follow-up are reported in Tables 2, 3 and 4; Fig. 3. Comparing baseline to follow-up examinations, in HFpEF, there was a significant deterioration of RV LAS at rest and during exercise-stress (*p* = 0.007 for both). In contrast, this was not observed in NCD with the only functional change being an improvement in LA LAS during exercise-stress (*p* = 0.028). There were no functional alterations in HFpEF patients who had undergone IASD implantation including preserved RV LAS.

**Table 2:** Cardiovascular magnetic resonance imaging follow-up.

Variable	NCD baseline	NCD follow-up	Significance *p*
**Laboratory testing**			
NTproBNP	55 (37, 111)	81 (65, 138)	**0.013**
**Echocardiography**			
E/e’	9.6 (8.6, 12.0)	10.1 (8.6, 12.0)	0.333
TAPSE	21.8 (19.1, 26.4)	23.6 (22.0, 28.5)	0.093
**Left ventricle**			
LV Mass	55.3 (48.1, 72.2)	52.3 (51.4, 62.4)	**0.015**
LV EDV	65.9 (54.9, 80.3)	70.8 (61.3, 86.2)	0.374
LV ESV	19.6 (13.0, 23.3)	23.1 (18.0, 29.5)	0.515
LV SV	45.1 (40.0, 59.2)	47.5 (44.3, 60.3)	0.260
LV EF	73.4 (63.9, 77.2)	70.1 (67.4, 71.7)	0.374
FT LV GLS	−26.6 (−23.4, −29.2)	−24.6 (−21.7, −28.6)	0.241
FT LV GCS	−37.2 (−40.0, −34.1)	−37.9 (−34.9, −41.8)	0.333
LV LAS rest	13.7 (13.1, 15.4)	14.2 (12.3, 16.5)	0.508
LV LAS stress	17.8 (13.9, 20.7)	16.6 (13.7, 20.8)	0.878
Septal Native T1	1314 (1288, 1335)	1347 (1294, 1376)	0.069
Septal ECV	24.9 (21.8, 26.9)	25.3 (23.6, 27.4)	0.342
**Left atrium**			
FT LA Es	27.1 (20.7, 29.6)	23.5 (19.6, 35.9)	0.799
FT LA Ee	12.2 (6.5, 17.2)	11.1 (7.9, 22.3)	0.139
FT LA E	12.7 (11.2, 16.8)	12.6 (7.5, 17.5)	0.575
LA LAS rest	21.0 (19.4, 23.3)	20.8 (17.2, 28.4)	0.646
LA LAS stress	20.5 (15.7, 28.9)	26.5 (20.9, 36.8)	**0.028**
**Right ventricle**			
RV EDV	64.6 (48.4, 76.6)	65.5 (59.2, 85.1)	0.214
RV ESV	22.4 (16.1, 28.7)	25.2 (19.1, 33.5)	0.110
RV SV	42.5 (34.8, 48.8)	40.8 (39.7, 51.2)	0.314
RV EF	65.3 (60.7, 70.9)	65.2 (56.0, 67.1)	0.767
RV LAS rest	18.3 (16.8, 22.2)	16.9 (13.9, 20.2)	0.333
RV LAS stress	18.9 (15.1, 23.3)	19.2 (10.9, 25.9)	0.878

TAPSE: tricuspid annular plane systolic excursion, LV: left ventricular, EDV/ESV: end-diastolic/-systolic volume, SV: stroke volume, EF: ejection fraction, FT: Feature-Tracking, GLS/GCS: Global longitudinal/circumferential strain, LAS: long axis strain, ECV: extracellular volume, LA: left atrium, Es/e/a: reservoir/conduit/booster pump function, RV: right ventricle. Bold p-values indicate statistical significance below 0.05. Volumes are given in ml/m² BSA, mass in g/m² BSA, strain/EF/ECV in %, T1 in ms, NTproBNP in pg/ml and TAPSE in mm.

**Table 3:** Cardiovascular magnetic resonance imaging follow-up.

Variable	HFpEF baseline	HFpEF follow-up	Significance *p*
**Laboratory testing**			
NTproBNP	209 (80, 320)	225 (86, 344)	0.646
**Echocardiography**			
E/e’	11.5 (10.3, 13.5)	9.9 (7.7, 13.1)	0.203
TAPSE	23.6 (21.0, 28.8)	24.6 (20.4, 28.0)	0.878
**Left Ventricle**			
LV Mass	52.2 (47.9, 62.1)	48.3 (45.2, 52.9)	**0.022**
LV EDV	65.3 (60.7, 75.7)	68.9 (57.5, 83.4)	0.959
LV ESV	16.7 (14.3, 24.5)	17.6 (12.7, 30.0)	0.285
LV SV	49.3 (44.3, 55.1)	49.3 (40.6, 54.5)	0.721
LV EF	75.8 (66.5, 77.2)	72.3 (62.6, 75.8)	0.445
FT LV GLS	−27.2 (−21.2, −30.4)	−26.0 (−23.1, −29.7)	0.878
FT LV GCS	−37.4 (−32.2, −41.2)	−39.4 (−34.0, −42.8)	0.575
LV LAS rest	13.8 (11.5, 15.5)	14.9 (13.6, 17.2)	0.059
LV LAS stress	15.6 (12.9, 17.5)	16.7 (12.5, 19.3)	0.959
Septal Native T1	1302 (1264, 1334)	1329 (1304, 1377)	**0.022**
Septal ECV	25.6 (24.4, 29.2)	27.3 (25.7, 29.5)	0.221
**Left Atrium**			
FT LA Es	22.8 (13.9, 24.6)	15.6 (12.1, 28.4)	0.386
FT LA Ee	11.0 (6.5, 13.5)	8.1 (6.2, 13.6)	0.959
FT LA Ea	10.5 (6.2, 13.5)	8.8 (4.7, 14.8)	0.285
LA LAS rest	12.8 (8.5, 20.9)	20.0 (8.9, 22.3)	0.285
LA LAS stress	18.3 (13.2, 20.5)	19.5 (16.8, 25.1)	0.114
**Right Ventricle**			
RV EDV	65.0 (58.1, 71.7)	67.4 (59.2, 75.8)	0.508
RV ESV	20.0 (16.4, 23.0)	21.3 (17.4, 24.7)	0.059
RV SV	45.6 (38.7, 51.3)	45.0 (41.8, 48.2)	0.721
RV EF	69.5 (63.7, 74.9)	68.0 (64.5, 71.4)	0.139
RV LAS rest	26.3 (20.9, 30.3)	19.6 (17.7, 25.2)	**0.007**
RV LAS stress	24.2 (21.2, 27.3)	18.6 (13.8, 22.4)	**0.007**

TAPSE: tricuspid annular plane systolic excursion, LV: left ventricular, EDV/ESV: end-diastolic/-systolic volume, SV: stroke volume, EF: ejection fraction, FT: Feature-Tracking, GLS/GCS: Global longitudinal/circumferential strain, LAS: long axis strain, ECV: extracellular volume, LA: left atrium, Es/e/a: reservoir/conduit/booster pump function, RV: right ventricle. Bold p-values indicate statistical significance below 0.05. Volumes are given in ml/m² BSA, mass in g/m² BSA, strain/EF/ECV in %, T1 in ms, NTproBNP in pg/ml and TAPSE in mm.

**Table 4:** Cardiovascular magnetic resonance imaging follow-up.

Variable	HFpEF IASD baseline	HFpEF IASD follow-up	Significance *p*
**Laboratory testing**			
NTproBNP	230 (141, 998)	267 (190, 431)	0.917
**Echocardiography**			
E/e’	12.8 (9.1, 12.9)	9.3 (7.7, 10.5)	0.173
TAPSE	25.6 (23.1, 28.5)	26.7 (19.4, 30.9)	0.917
**Left Ventricle**			
LV Mass	60.0 (47.0, 67.5)	46.5 (38.3, 60.1)	**0.028**
LV EDV	59.0 (51.6, 74.4)	65.7 (42.8, 81.2)	0.917
LV ESV	17.1 (14.7, 26.4)	17.8 (12.2, 28.8)	0.753
LV SV	44.6 (33.0, 51.0)	49.3 (29.0, 54.2)	0.917
LV EF	71.6 (58.4, 75.8)	67.3 (63.8, 75.0)	0.753
FT LV GLS	−23.4 (−20.8, −26.8)	−24.3 (−21.7, −25.3)	0.917
FT LV GCS	−40.8 (−28.4, −43.5)	−39.0 (−35.7, −41.7)	0.753
LV LAS rest	12.3 (7.5, 13.9)	12.7 (11.7, 16.9)	0.249
LV LAS stress	13.6 (11.6, 17.0)	14.7 (12.8, 18.8)	0.463
Septal Native T1	1344 (1289, 1395)	1287 (1064, 1472)	0.600
Septal ECV	25.0 (24.9, 27.0)	26.5 (25.6, 27.2)	0.345
**Left Atrium**			
FT LA Es	20.7 (14.4, 24.5)	20.9 (12.8, 25.5)	0.917
FT LA Ee	8.5 (7.9, 12.7)	9.5 (6.3, 15.4)	0.753
FT LA Ea	10.0 (6.2, 13.7)	10.3 (5.0, 13.7)	0.753
LA LAS rest	12.1 (8.2, 16.4)	14.1 (11.5, 21.1)	0.345
LA LAS stress	13.0 (8.9, 18.5)	15.3 (13.7, 24.0)	0.075
**Right Ventricle**			
RV EDV	65.1 (51.3, 81.4)	76.3 (52.5, 100.9)	0.345
RV ESV	23.2 (16.9, 29.2)	28.3 (22.3, 41.0)	0.116
RV SV	44.1 (28.9, 55.8)	41.0 (32.8, 69.0)	0.917
RV EF	64.8 (60.2, 68.9)	61.7 (53.4, 67.8)	0.345
RV LAS rest	18.0 (16.8, 20.9)	18.2 (15.3, 19.9)	0.600
RV LAS stress	21.6 (19.2, 27.0)	16.1 (7.6, 22.2)	0.075

TAPSE: tricuspid annular plane systolic excursion, LV: left ventricular, EDV/ESV: end-diastolic/-systolic volume, SV: stroke volume, EF: ejection fraction, FT: Feature-Tracking, GLS/GCS: Global longitudinal/circumferential strain, LAS: long axis strain, ECV: extracellular volume, LA: left atrium, Es/e/a: reservoir/conduit/booster pump function, RV: right ventricle. Bold p-values indicate statistical significance below 0.05. Volumes are given in ml/m² BSA, mass in g/m² BSA, strain/EF/ECV in %, T1 in ms, NTproBNP in pg/ml and TAPSE in mm.

**Table 5:** Cardiovascular magnetic resonance imaging follow-up.

Variable	NCD follow-up	HFpEF follow-up	HFpEF IASD follow-up	NCD vs. HFpEF	NCD vs. IASD	HFpEF vs. IASD
**Laboratory testing**						
NTproBNP	81 (65, 138)	225 (86, 344)	267 (190, 431)	0.105	**0.022**	0.492
**Echocardiography**						
E/e’	10.1 (8.6, 12.0)	9.9 (7.7, 13.1)	9.3 (7.7, 10.5)	0.912	0.368	0.562
TAPSE	23.6 (22.0, 28.5)	24.6 (20.4, 28.0)	26.7 (19.4, 30.9)	0.720	0.864	0.635
**Left Ventricle**						
LV Mass	52.3 (51.4, 62.4)	48.3 (44.5, 54.7)	46.5 (38.3, 60.1)	**0.035**	0.147	0.635
LV EDV	70.8 (61.3, 86.2)	68.9 (52.3, 80.8)	65.7 (42.8, 81.2)	0.739	0.368	0.562
LV ESV	23.1 (18.0, 29.5)	17.6 (12.8, 28.6)	17.8 (12.2, 28.8)	0.529	0.428	0.958
LV SV	47.5 (44.3, 60.3)	49.3 (40.0, 53.3)	49.3 (29.0, 54.2)	0.796	0.713	0.713
LV EF	70.1 (67.4, 71.7)	72.3 (62.6, 75.8)	67.3 (63.8, 75.0)	0.529	0.635	0.792
FT LV GLS	−24.6 (−21.7, −28.6)	−24.6 (−22.7, −27.3)	−24.3 (−21.7, −25.3)	0.739	0.713	0.263
FT LV GCS	−37.9 (−34.9, −41.8)	−39.4 (−34.6, −41.9)	−39.0 (−35.7, −41.7)	1.000	0.875	0.958
LV LAS rest	14.2 (12.3, 16.5)	14.6 (12.6, 17.0)	12.7 (11.7, 16.9)	0.315	0.792	0.428
LV LAS stress	16.6 (13.7, 20.8)	15.6 (12.7, 19.1)	14.7 (12.8, 18.8)	0.796	0.562	0.958
Septal Native T1	1347 (1294, 1376)	1329 (1304, 1377)	1287 (1064, 1472)	0.968	0.272	0.147
Septal ECV	25.3 (23.6, 27.4)	27.3 (25.7, 29.5)	26.5 (25.6, 27.2)	0.133	0.328	0.263
**Left Atrium**						
FT LA Es	23.5 (19.6, 35.9)	15.6 (12.1, 28.4)	20.9 (12.8, 25.5)	0.218	0.313	0.875
FT LA Ee	11.1 (7.9, 22.3)	8.1 (6.2, 13.6)	9.5 (6.3, 15.4)	0.280	0.368	0.958
FT LA Ea	12.6 (7.5, 17.5)	8.8 (4.7, 14.8)	10.3 (5.0, 13.7)	0.247	0.368	0.958
LA LAS rest	20.8 (17.2, 28.4)	20.0 (8.9, 22.3)	14.1 (11.5, 21.1)	0.353	0.118	0.713
LA LAS stress	26.5 (20.9, 36.8)	19.5 (16.8, 25.1)	15.3 (13.7, 24.0)	0.063	**0.042**	0.368
**Right Ventricle**						
RV EDV	65.5 (59.2, 85.1)	67.4 (59.2, 75.8)	76.3 (52.5, 100.9)	0.912	0.713	0.492
RV ESV	25.2 (19.1, 33.5)	21.3 (17.4, 24.7)	28.3 (22.3, 41.0)	0.481	0.635	0.181
RV SV	40.8 (39.7, 51.2)	45.0 (41.8, 48.2)	41.0 (32.8, 69.0)	0.393	0.875	0.635
RV EF	65.2 (56.0, 67.1)	68.0 (64.5, 71.4)	61.7 (53.4, 67.8)	0.075	0.713	0.147
RV LAS rest	16.9 (13.9, 20.2)	19.6 (17.7, 25.2)	18.2 (15.3, 19.9)	0.315	0.792	0.428
RV LAS stress	19.2 (10.9, 25.9)	18.6 (13.8, 22.4)	16.1 (7.6, 22.2)	0.912	0.220	0.428

TAPSE: tricuspid annular plane systolic excursion, LV: left ventricular, EDV/ESV: end-diastolic/-systolic volume, SV: stroke volume, EF: ejection fraction, FT: Feature-Tracking, GLS/GCS: Global longitudinal/circumferential strain, LAS: long axis strain, ECV: extracellular volume, LA: left atrium, Es/e/a: reservoir/conduit/booster pump function, RV: right ventricle. Bold p-values indicate statistical significance below 0.05. Volumes are given in ml/m² BSA, mass in g/m² BSA, strain/EF/ECV in %, T1 in ms, NTproBNP in pg/ml and TAPSE in mm.

### Follow-up

At follow-up, compared to NCD, LA LAS during exercise-stress was impaired in IASD patients (*p* = 0.042) whilst HFpEF patients showed a strong statistical trend (*p* = 0.063), Table 5.

### Outcome

Within both the HFpEF (2x tachyarrhythmia (TAA), 1x ICD) and NCD (1x TAA, 2xPTCA) group, 3 cardiovascular events were noted, in the IASD group 2 events (2xTAA). Neither RV function at follow-up as appreciated from RV LAS at rest (HR 0.99, 95% CI 0.87–1.13, *p* = 0.884) and during exercise-stress (HR 0.96, 95% CI 0.88–1.06, *p* = 0.441) nor the absolute change from baseline to follow-up for RV LAS at rest (HR 0.96, 95% CI 0.86–1.09, *p* = 0.544) or during exercise-stress (HR 1.00, 95% CI 0.93–1.07, *p* = 0.893) were associated with CVE 4 years following initial study participation. In contrast, LA function at follow-up was associated with CVE including FT Es/Ea and LA LAS at rest and during exercise-stress, Table 6.

**Table 6:** Cardiac function and events.

Variable	Hazard ratio	Significance *p*
**Left ventricle**		
LV EF	0.89 (0.80–0.99)	**0.039**
FT LV GLS	1.21 (0.97–1.52)	0.093
FT LV GCS	1.10 (0.94–1.28)	0.219
LV LAS rest	0.92 (0.69–1.24)	0.584
LV LAS stress	0.82 (0.65–1.02)	0.076
Septal Native T1	1.00 (1.00–1.01)	0.547
Septal ECV	1.02 (0.80–1.31)	0.863
**Left atrium**		
FT LA Es	0.88 (0.79–0.97)	**0.010**
FT LA Ee	0.86 (0.71–1.04)	0.115
FT LA Ea	0.79 (0.67–0.94)	**0.006**
LA LAS rest	0.84 (0.74–0.95)	**0.006**
LA LAS stress	0.86 (0.77–0.97)	**0.011**
**Right ventricle**		
RV EF	1.03 (0.93–1.14)	0.624
RV LAS rest	0.99 (0.87–1.13)	0.884
RV LAS stress	0.96 (0.88–1.06)	0.441

LV: left ventricular, EF: ejection fraction, FT: Feature-Tracking, GLS/GCS: Global longitudinal/circumferential strain, LAS: long axis strain, ECV: extracellular volume, LA: left atrium, Es/e/a: reservoir/conduit/booster pump function, RV: right ventricle. Bold p-values indicate statistical significance below 0.05.

## Discussion

The present results from the follow-up scans of the HFpEF-Stress Trial provide further insights into the course of pathophysiological alterations in HFpEF. First, HFpEF patients showed a significant decline in RV longitudinal deformation at rest and during exercise-stress. In contrast NCD or IASD patients did not show cardiac functional deterioration from baseline to follow-up. Second, NCD patients on the other hand showed improvement in LA function during exercise-stress. Last, LA but not RV function was associated with cardiovascular events 4 years after baseline participation.

In patients with chronic dyspnoea, exercise-induced pulmonary hypertension (PH) is – in the absence of PH at rest - associated with worse outcome. This finding emerged independent of both pre- and post-capillary contributions^23^. Elevated PCWP in HFpEF may lead to PVD and increased PVR^24^ which in turn is associated with impaired RV contractility^10^. Indeed, beyond LV dysfunction, impaired RV reserve during exercise is a distinct feature in HFpEF^25^. Recent results from the Reduce-LAP trials highlight that patients with latent PVD show worse outcome following IASD implantation^8^. This can at least in parts be attributed to impaired RV functional reserve challenged by volume overload due to shunt flow. Impaired RV functional reserve subsequently results in reduced LA and LV filling leading to reduced cardiac output^10^.

At baseline, HFpEF patients showed increased RV LAS compared to NCD or IASD. The present follow-up demonstrates a decrease in RV deformation both at rest and during exercise-stress from baseline to follow-up in HFpEF but not NCD or IASD. Indeed, at baseline, HFpEF patients showed higher PVR compared to HFpEF patients selected for IASD. Consequently, increased RV LAS at baseline may be a sign of early compensation for latent PVD with deterioration during disease progression.

Intriguingly, these findings were made by longitudinal deformation only, whilst volumetric changes were not observed. This again may highlight the sensitivity of longitudinal deformation over volumetric analysis to uncover masked pathophysiological changes of the heart^10,26^. However, during later stages of disease in overt HFpEF right ventricular deterioration becomes apparent on volumetric analyses as well^27^. Noteworthy, a significant deterioration of RV longitudinal deformation was not observed in the HFpEF IASD subgroup. Reduction of PCWP by shunt volume may have attenuated progress in latent PVD and PVR. Indeed, in IASD, there was a statistical trend for deterioration of RV LAS during exercise-stress only. This may further indicate that progress in RV functional deterioration was attenuated by IASD implantation becoming apparent with a statistical trend only by exercise-stress testing. Subgroup analyses from the Reduce LAP HF II trial demonstrated that only patients in the absence of latent PVD may benefit from IASD implantation^8^. The finding of RV functional deterioration in HFpEF as opposed to IASD may thus also be influenced by the difference in PVR at baseline. Strikingly, RV LAS but not TAPSE quantified functional deterioration or RV function during follow-up. On the one hand this may root in methodology with acoustic windows tending to be more limited in patients presenting with exertional dyspnoea e.g. due to obesity^28^. Indeed, CMR has a class I recommendation in HF patients with poor acoustic windows^1^. Furthermore, visualisation of the RV tends to be more challenging compared to the LV. On the other hand, RV LAS may emerge superior for RV functional quantification. Echocardiographic TAPSE showed distinctly lower correlation to CMR derived RV EF compared to RV GLS^29^. Further data indicates superiority of strain for prognostic evaluation compared to TAPSE including following inferior acute myocardial infarction^30^ or aortic valve replacement^31,32^. In line, RV LAS added incremental value to TAPSE in HCM. This may indicate that both measurements of RV function are rather complementary than interchangeable^33^.

Notwithstanding, LA but not RV function was associated with cardiovascular events. First, most of the events in the HFpEF and IASD group were linked to cardiac congestion induced by tachyarrhythmia. In contrast 2 out of 3 events in the NCD group were linked to coronary artery disease. In that regard, association of LA rather than RV function can - in parts - be interpreted due to the nature of cardiovascular events. Secondly, RV systolic dysfunction as RVEF < 47% has been reported to be associated with death and/or heart failure hospitalization^34^. In the present follow-up population, none presented with an RVEF below 47%. Lastly, more than half of all HFpEF patients had been identified by exercise-stress thresholds only^13^. Consequently, an average of 3 years between baseline and follow-up scan as well as a total of 4 years follow-up for event identification from baseline recruitment may be insufficient for full evaluation of the long-term impact of RV deterioration and heart failure hospitalisation/mortality.

### Study limitations

Conclusions from the rescan follow-up of the monocentric HFpEF Stress Trial are based on limited patient numbers. Therefore, conclusions made must be considered hypothesis-generating sparking further research rather than final conclusions for the pathophysiology of HFpEF. Especially the low number of patients with IASD limits findings to a hypothesis-generating nature. Notwithstanding, identifying significant statistical changes within this small population underlines their prominence.

## Conclusion

RV functional deterioration may be a pathophysiological feature of progress in early-stage HFpEF as opposed to NCD and HFpEF treated with IASD. Longitudinal deformation imaging may emerge more sensitive to unmask early changes as opposed to volumetric assessments. Further larger multi-centre studies are warranted to verify these hypothesis-generating results.

## Data Availability

The data underlying the findings is available at the imaging database of the University Hospital Goettingen and access will be granted to researchers that meet the criteria for access upon formal request from corresponding author.
