# Author Correction: Insights from serial cardiovascular magnetic resonance imaging show early progress in diastolic dysfunction relates to impaired right ventricular deformation

**DOI:** 10.1038/s41598-025-93248-2

**Published:** 2025-03-10

**Authors:** Sören J. Backhaus, Alexander Schulz, Torben Lange, Simon F. Rösel, Lennart S. Schmidt-Schweda, Shelby Kutty, Johannes T. Kowallick, Julia Treiber, Andreas Rolf, Samuel Sossalla, Gerd Hasenfuß, Andreas Schuster

**Affiliations:** 1 Department of Cardiology, Campus Kerckhoff of the Justus-Liebig-University Giessen, Kerckhoff-Clinic, Bad Nauheim, Germany; 2 German Center for Cardiovascular Research (DZHK), Partner Site Rhine-Main, Bad Nauheim, Germany; 3 Department of Cardiology and Angiology, Medical Clinic I, University Hospital Giessen, Justus-Liebig-University Giessen, Giessen, Germany; 4 Department of Cardiology and Pneumology, University Medical Center Göttingen, Georg-August University, Göttingen, Germany; 5 German Center for Cardiovascular Research (DZHK), Partner Site Lower Saxony, Göttingen, Germany; 6 Department of Medicine, Cardiovascular Division, Beth Israel Deaconess Medical Center, Harvard Medical School, Boston, USA; 7 Taussig Heart Center, Johns Hopkins Hospital, Baltimore, MD USA; 8 FORUM Radiology, Rosdorf, Germany; 9 FORUM Cardiology, An der Ziegelei 1, 37124 Rosdorf, Germany

Correction to: *Scientific Reports* 10.1038/s41598-025-87032-5, published online 10 February 2025

The original version of this Article contained an error in the spelling of the author Gerd Hasenfuß which was incorrectly given as Gerd Hasenuß.

The original Article has been corrected.

